# Evaluating the impact of sample storage, handling, and technical ability on the decay and recovery of SARS-CoV-2 in wastewater

**DOI:** 10.1371/journal.pone.0270659

**Published:** 2022-06-24

**Authors:** Rachelle E. Beattie, A. Denene Blackwood, Thomas Clerkin, Carly Dinga, Rachel T. Noble

**Affiliations:** Department of Earth, Marine, and Environmental Sciences, Institute of Marine Science, University of North Carolina at Chapel Hill, Morehead City, North Carolina, United States of America; University of Helsinki: Helsingin Yliopisto, FINLAND

## Abstract

Wastewater based epidemiology (WBE) is useful for tracking and monitoring the level of disease prevalence in a community and has been used extensively to complement clinical testing during the current COVID-19 pandemic. Despite the numerous benefits, sources of variability in sample storage, handling, and processing methods can make WBE data difficult to generalize. We performed an experiment to determine sources of variability in WBE data including the impact of storage time, handling, and processing techniques on the concentration of SARS-CoV-2 in wastewater influent from three wastewater treatment plants (WWTP) in North Carolina over 19 days. The SARS-CoV-2 concentration in influent samples held at 4°C did not degrade significantly over the 19-day experiment. Heat pasteurization did not significantly impact the concentration of SARS-CoV-2 at two of the three WWTP but did reduce viral recovery at the WWTP with the smallest population size served. On each processing date, one filter from each sample was processed immediately while a replicate filter was frozen at -80°C. Once processed, filters previously frozen were found to contain slightly higher concentrations (<0.2 log copies/L) than their immediately processed counterparts, indicating freezing filters is a viable method for delayed quantification and may even improve recovery at WWTP with low viral concentrations. Investigation of factors contributing to variability during sample processing indicated that analyst experience level contributed significantly (*p*<0.001) to accepted droplet generation while extraction efficiency and reverse transcription efficiency contributed significantly (*p*<0.05) to day-to-day SARS-CoV-2 variability. This study provides valuable practical information for minimizing decay and/or loss of SARS CoV-2 in wastewater influent while adhering to safety procedures, promoting efficient laboratory workflows, and accounting for sources of variability.

## Introduction

Traditional epidemiology relies on clinical testing data to determine disease occurrence and patterns in a specific region. However, in areas where clinical testing is inaccessible or unavailable or in regions with high numbers of asymptomatic disease carriers, wastewater-based epidemiology (WBE) for human pathogens provides an alternative to quantify disease trends at the population level. Quantifying concentrations of pathogens of interest in wastewater provides a pooled assessment of disease prevalence in a community in real time, complementing clinical case counts [1]. This approach has been employed globally throughout the ongoing SARS-CoV-2 pandemic to monitor patterns in viral load and to aid in public health decisions [2–4] as concentrations of SARS-CoV-2 in wastewater have been shown to be highly correlative with and predictive of clinical patterns in disease occurrence [5–8]. Despite these benefits, quantification of pathogens of interest in wastewater can be difficult. Sample matrix complexity and variability in laboratory processing procedures have made comparisons between sewersheds and processing laboratories difficult to interpret which increases the risk of false positive and false negative errors [9]. In order for WBE measurements to be useful for public health decisions and management globally, consideration of sources of variability in viral recovery needs to be addressed.

Viral decay and recovery are two major challenges associated with sewershed comparisons of WBE for human pathogens. At the wastewater treatment plant (WWTP) level, differences in population size, flow, and influent characteristics contribute to variability in viral decay and recovery between WWTP [1, 9]. The influence of population size on the sewage system should be considered as this directly impacts critical infrastructure maintenance, funding allocated for repairs, detection of sewage system leaks, and, when sewage infrastructure has been damaged, the amount of inflow and infiltration that enters the sewage system. Inflow and infiltration of stormwater, seawater or ground water alters influent characteristics impacting viral decay and recovery. Additionally, sample storage and pre-processing steps including temperature, time, and handling may impact the concentration of virus recovered [9, 10]. A significant amount of research for WBE of pathogens has focused on developing best practices for viral concentration, extraction, and quantification [11–13]; however, a better understanding of sample storage and pre-processing steps is necessary to ensure consistent recovery regardless of the downstream methods used. Previous research in this area has also been conducted using low temporal resolution, typically measuring concentrations of viral pathogens at 24 hours, 3 days, 7 days, and 14 days with no measurements in the interim timeframes [10, 14].

Here, we performed a series of experiments on wastewater influent samples from three separate WWTP in the state of North Carolina. We expand upon previous studies in this area by using a high frequency temporal analysis approach to assess variability in SARS-CoV-2 N1 and N2 gene decay and recovery. Daily, for 19 days, we measured concentrations of SARS-CoV-2 in wastewater influent providing a high-resolution account of any changes induced by various sample handling, storage, and processing techniques. Additionally, the population size served and wastewater parameters vary at each of the three WWTP sampled, and the impact of these factors on viral decay, loss, and recovery was evaluated. This study was designed to evaluate the impact of a) storage time; b) heat pasteurization; c) sample source; and d) freeze-thaw processes; and e) technical ability on the concentration of SARS-CoV-2 in wastewater influent held at 4°C. Additionally, the impacts of extraction efficiency, reverse transcription efficiency, and inhibition on viral concentration variability were assessed. Droplet digital PCR (ddPCR) was used in this study for the detection of RNA from SARS-CoV-2 in wastewater influent based on the amplification of two regions of the Nucleocapsid gene of SARS-CoV-2, N1 and N2, and an array of exogenous controls following the digital MIQE guidelines (Minimum Information for Publication of Quantitative Digital PCR Experiments) [15]. The results from this work provide a high-resolution account of sample handling and pre-processing factors impacting viral decay and recovery. Additionally, this work identifies pinch points during which laboratory teams can stop and restart analysis without significant viral decay and/or loss. Together these data will help guide laboratories on the best methods for reducing viral decay in wastewater samples used for WBE of pathogens.

## Materials and methods

### Sample collection and pre-processing

**Fig 1:**
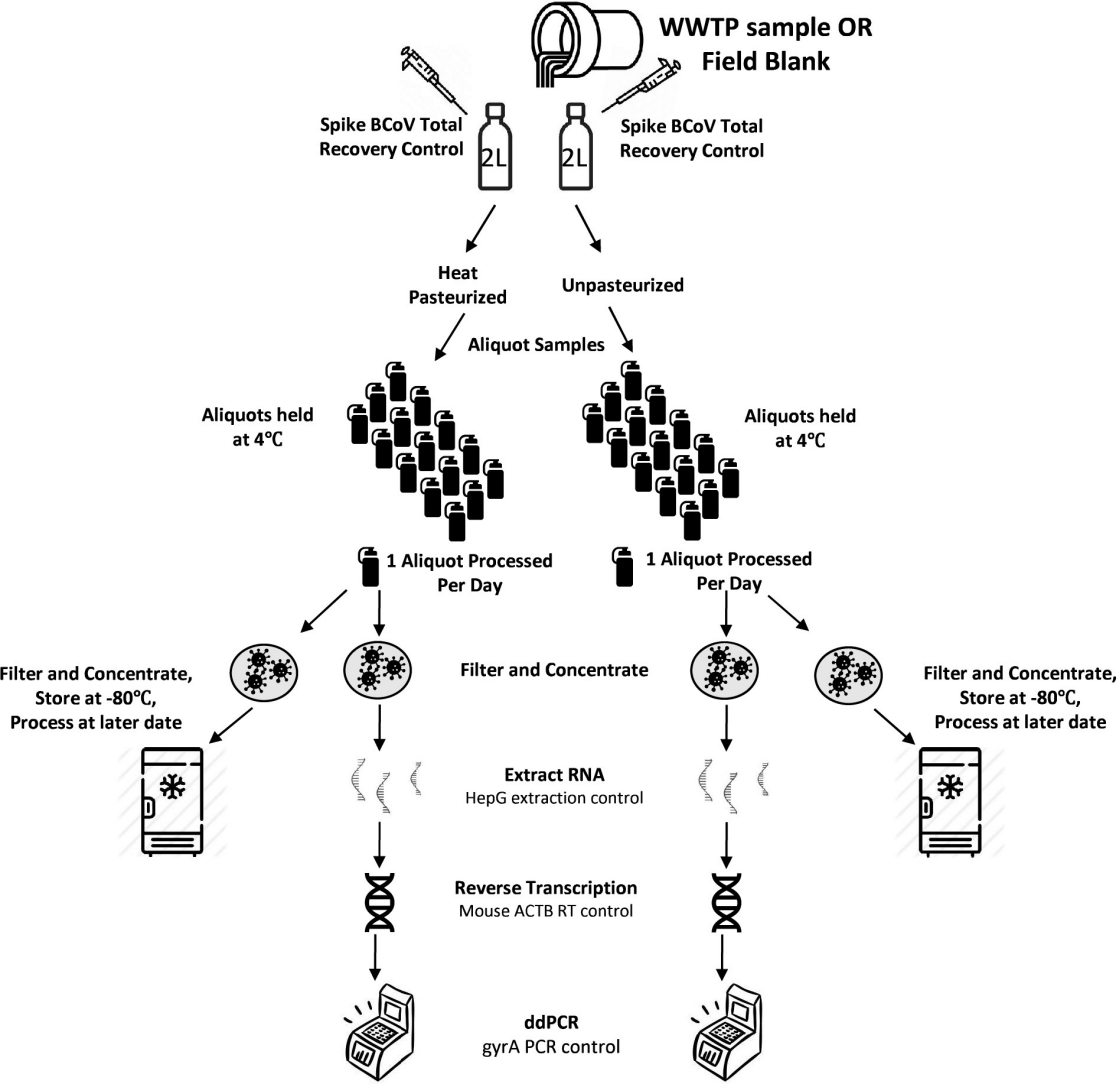
Overview of sample collection and processing with controls for each step outlined.

An overview of the methods and processing flow used in this study can be viewed in Fig 1. Wastewater samples used for this study were collected with WWTP permission as part of the North Carolina Department of Human and Health Services wastewater surveillance network with support from the United States Centers for Disease Control National Wastewater Surveillance System. Influent wastewater from three separate WWTP in North Carolina was collected on August 9, 2021, at approximately 8 a.m. (see Table 1 for WWTP details). At each WWTP, influent was subsampled in a sterile manner from a 24-hour flow-weighted composite sample. Each catchment used an autosampler to collect influent samples per million gallons of flow. Composite influent subsamples were transferred to sterile 2L polypropylene sample bottles at each WWTP. Duplicate 2L field blanks were filled with filter-sterilized 1X phosphate buffered saline (PBS) at UNC Institute of Marine Sciences (IMS, Morehead City, NC) and transported to one of the three WWTP. To address environmental contamination, field blank samples were uncapped at the facility next to the composite sampler to introduce the sample to the environment and then recapped.

**Table 1:** Permitted flow, population size served, and 24-hour influent and effluent flow on the day of sample collection for the three WWTP in this study. See S1 Table in S1 File for metric conversions of million gallons per day (MGD).

WWTP ID	Permitted Influent Flow (MGD)	Estimated Population Served [16]	24 Hour Influent Flow (MGD): August 9, 2021	24 Hour Effluent Flow (MGD): August 9, 2021	Total Suspended Solids (mg/L): August 9, 2021
A	21.0	151,589	15.8	15.0	253
B	75.0	550,000	46.98	45.831	288
C	10.5	67,743	8.64	14.548	156

Immediately after sample collection, each research analyst at one of the three WWTP thawed and spiked 2000 copies of pre-quantified Bovine Coronavirus (BCoV:MERECK Animal Health BOVILIS® Coronavirus Calf Vaccine, PBS Animal Health Massillon, OH) containing approximately 10 copies/μL (same lot and batch) into each sample at precisely the same time to serve as a Process Recovery Control. Samples were then transported back to IMS on wet ice (time from collection to processing <5 hours). Upon arrival, one 2L sample from each site was heat pasteurized in a water bath at 65˚C for 1 hour to reach an internal sample temperature of 56˚C for 30 minutes as recommended by the CDC [17]. The duplicate 2L sample was left unpasteurized and placed in mechanical refrigeration at 4˚C. All samples (pasteurized and unpasteurized) were mixed by inversion for 30 seconds and 90 mL was poured off into 19 sterile containers for each analysis point. One replicate of each sample type was left out for Day 0 concentration and processing while all other replicates were kept in mechanical refrigeration at 4˚C.

### Sample concentration and total nucleic acid extraction

Beginning on the day of sample collection (Day 0) and continuing daily thereafter, two 90 mL replicates from each WWTP and the field blank (pasteurized and unpasteurized) were removed from 4˚C and pH adjusted to 3.5 as confirmed by pH paper test strips by the addition of 400 μL of 10M HCl. Following the method of Katayama et al. [18], 1.8 mL of filter sterilized 1.25 M MgCl_2_ x 6 H_2_O (50X) was added to each 90 mL replicate for a final concentration of 25 mM. A 90 mL method blank (1X PBS) was also prepared daily and amended with HCl and MgCl_2_ as above. Samples were then mixed by inversion for 30 seconds and 40 mL of each sample was vacuum filtered to dryness through 0.45 μm Mixed Cellulose Ester (MCE) filter type GN-6 Metricel 47 mm disposable filter funnels (Pall Corp., Port Washington, NY) in duplicate. Filters were folded using sterile forceps and placed in a 2mL microcentrifuge tube. One filter was immediately submerged in 1 mL easyMag® Lysis buffer (bioMerieux, Durham, NC) containing 74 copies of the extraction recovery control, Armored RNA® containing hepatitis G sequence (HepG, Assurgen Austin, TX), and the other filter was stored at -80°C for future analysis. The lysed samples were incubated at room temperature for 10 minutes followed by total nucleic acid extraction using an automated magnetic particle analyzer (KingFisher™ Flex, Thermo Fisher Scientific, Waltham, MA) and easyMag® NucliSENS ® reagents (bioMerieux, Durham, NC) following the script provided in the supplementary information. Extractions were eluted in 100 uL buffer AE (QIAGEN, Germantown, MD). This process was repeated daily at precisely 08:00 for an additional 18 consecutive days (total of 19 timepoints).

### Reverse transcription and droplet digital PCR

Extracted RNA was immediately converted to cDNA using a two-step reverse transcription (RT) process. Of the 100 μL nucleic acid eluted, 25 μL was added to an RT mastermix containing 10 μL of SuperScript™ IV VILO™ cDNA synthesis mastermix (Thermo Fisher), 14 μL of nuclease free water, and 1 uL containing ~5 copies/μL of Mouse Normal Lung Total RNA (mouse lung, BioChain, Newark, CA) to determine the RT efficiency for a total reaction volume of 50 μL. This process inherently dilutes the extracted RNA 1:2. The RT thermal cycling conditions were as follows: 10 minutes at 25°C to anneal primers, 10 minutes at 50°C for the RT reaction, and 5 minutes at 85°C for enzyme inactivation. All RT reactions were performed in a C1000 Touch™ thermal cycler (BioRad Laboratories, Hercules, CA).

PCR mastermixes targeting the N1 and N2 regions of the nucleocapsid (N) gene of SARS-CoV-2 [19] and spiked controls were created by adding 0.9nM of the respective forward and reverse primers and 0.25nM of the respective probe (see S2 Table in S1 File for primer/probe sequences) to 12.5μL of ddPCR™ 2X Supermix for Probes (no dUTP, BioRad Laboratories), 5 μL cDNA template, and nuclease free water to a final reaction volume of 25 μL. Twenty μL of the PCR mastermix and sample were pipetted into sample wells of the DG8™ Cartridge (BioRad,) using a manual 8-channel pipette (L8-50XLS+, Rainin, Oakland, CA) followed by the addition of 70 μL of Droplet Generation Oil for Probes (Bio-Rad) to the oil wells. The cartridges were covered with DG8™ Gaskets (Bio-Rad) and processed in a Droplet Generator (Bio-Rad). The droplets were gently transferred to a semi-skirted 96-well PCR plate (mTEC, Eppendorf, Framingham, MA) using a manual 8-channel pipette. The PCR plate was sealed with pierceable foil (Bio-Rad) using a PX1™ PCR Plate Sealer (Bio-Rad).

The PCR plate was placed in a C1000 Touch™ Thermal Cycler and amplification was performed with the following temperature profile: 10 min at 95°C for initial denaturation, 40 cycles of 94°C for 30 s, and 55°C for 60 s with a ramp rate of 2.5˚ per second (annealing temperature for all primers/probes was 55°C), followed by 98°C for 10 min, then an indefinite hold at 4˚C. After PCR cycling was complete, the plate was placed in a QX200™ instrument (BioRad) and droplets were analyzed according to manufacturer’s instructions for 6-FAM™/VIC™ and 6-FAM™/HEX™. Data acquisition and analysis were performed with QuantaSoft™ v. 1.7 (Bio-Rad).

### Quality control

#### Limit of the Blank and Limit of Detection

The Limit of Blank (LOB) was determined using eight technical replicates of eight negative influent samples. The LOB was calculated as LOB = mean blank + 1.645(std dev blank). The Limit of Detection (LOD) was then estimated through a classical estimation of LOD = LOB + 2(std dev) as defined by Armbruster et al., 2008 [20]. The LOD was determined to be 1070 copies/L and 330 copies/L for N1 and N2, respectively.

#### Negative controls

One negative extraction control (NEC) was included with daily extractions to verify the absence of cross-contamination. NECs consisted of an MCE filter with no sample and were processed under the same conditions as the other samples. No RT (NRT) controls used 5 μL of positive template in the absence of RT in the RT mastermix and served to confirm that only RNA is transcribed; NRT controls were included with each assay plate. No template controls (NTCs) used 5 μL of nuclease free water in place of cDNA template and were added to each assay plate to verify the absence of contamination in the master mix. No NTCs in this study were positive. NEC, NRT and a minimum of 4 NTC controls were run on every assay plate. Filtration method blanks and field blank samples were also incorporated into the daily analysis to address any concerns of sample contamination.

#### Positive controls

A heat inactivated SARS-CoV-2 positive RNA control, strain designation 2019-nCoV/USA-WA1/2020 (ATCC Bethesda, MD), was included with every RT and PCR plate. Positive controls were performed in duplicate and wells were merged for analysis.

A total processing and recovery control, Bovine Coronavirus [21], was added to the untreated wastewater prior to filtration to measure the amount of RNA loss throughout the entire process. An extraction control, hepatitis G [22], was added prior to the RNA extraction step to monitor losses during the extraction process. An RT control, mouse normal lung total RNA, was added prior to the RT step and mouse actin beta was measured as a means to determine RT efficiency. PCR efficiency and inhibition was measured by the addition of 140 copies of a haloalkaliphilic archaeon and quantification of the archaeon’s gyr *a* gene to address matrix inhibition. Primers and probes for the inhibition assay (gyr *a*) were kindly provided by Josh Steele (Southern California Coastal Water Research Project) and have not been published. Primers and probes were synthesized by LGC Biosearch Technologies (Novato, CA) with the exception of mouse actin beta (VIC®/MGB Probe, Primer Limited, Life Technologies). All assay conditions were previously optimized and established for the Noble laboratory by using manufacturer recommendations for reagent concentrations and a thermal gradient to determine the best annealing temperature. A thermal gradient ranging from 53–63°C was run on each positive control in duplicate and the temperature with the best separation of positive and negative droplets was selected as the annealing temperature for each assay.

#### Inhibition assessment

Reverse transcription and PCR inhibition were assessed for each sample by evaluating the measured concentration of the RT control (mouse actin beta) and the inhibition control (gyr *a* gene of haloalkaliphilic archaeon) added to each sample. Samples were considered inhibited if the control concentration was more than 1 standard deviation from the concentration added to each sample. No samples in this study exhibited RT or PCR inhibition.

#### Droplet Acceptance Criteria

The fluorescence amplitude threshold, distinguishing positive from negative droplets for ddPCR, was set manually by the analyst at the midpoint between the average baseline fluorescence amplitude of the negative droplet cluster and the positive droplet cluster [23]. The same threshold was applied to all the wells of one PCR plate. Samples were quantified in duplicate and replicate wells were merged. Results were excluded if the total number of accepted droplets was <20,000 or the average fluorescence amplitudes of positive or negative droplets were clearly different from those of the other wells on the plate. The QuantaSoft software uses the Poisson distribution to quantitate the concentration of targets based on the numbers of positive and accepted droplets in each well. This concentration was then then transferred to an in-house developed spreadsheet to normalize concentrations to target copies per 1L of wastewater by correcting for dilution factors, elution volume, and original volume of sample filtered (see, S1 Equation in S1 File). Samples were considered positive and quantifiable for the specified target if the positive droplet count was equal to or greater than three and the NTCs contained no more than 1 positive droplet for the four combined wells run on each plate.

### Data analysis

Based on the accepted droplet count required for the Poisson distribution in ddPCR (see Droplet Acceptance Criteria above), 6 of 152 samples were excluded from analysis. Statistical analyses were performed in GraphPad Prism v.9.2.0 (GraphPad Software, San Diego, CA, USA) or R open source computing software v.4.1.1 [24]. Shapiro-Wilks tests were performed to assess the normality of the data with *p* values <0.05 considered non-normal distributions [25]. Data from all WWTP and field blanks fell within the normal distribution with low kurtosis (S3 Table in S1 File), and thus raw data values were used in all subsequent analyses. Linear regression was used to model the relationship of SARS-CoV-2 N1 and N2 concentration over time [26]. Analysis of variance (ANOVA) followed by multiple comparisons was used to assess inter-sample variation while unpaired t-tests were used to assess intra-sample variation. Distance-based redundancy analysis was performed using the vegan package in R [27] to identify which, if any, factors in the sample processing procedure impacted variability in the concentration of SARS-CoV-2 N1 and N2. All data visualizations were created in GraphPad.

## Results

### Impact of holding time and heat pasteurization on SARS-CoV-2 concentration in wastewater

A variety of factors can influence variation in the quantification of viral pathogens within wastewater. Here, we assessed sample storage and handling procedures on the decay rate and recovery concentration of SARS-CoV-2 from three WWTP in North Carolina. Replicate samples were analyzed daily for 19 days to provide high resolution data on the quantification and decay of SARS-CoV-2 providing guidance for pre-processing and storage methods.

**Fig 2:**
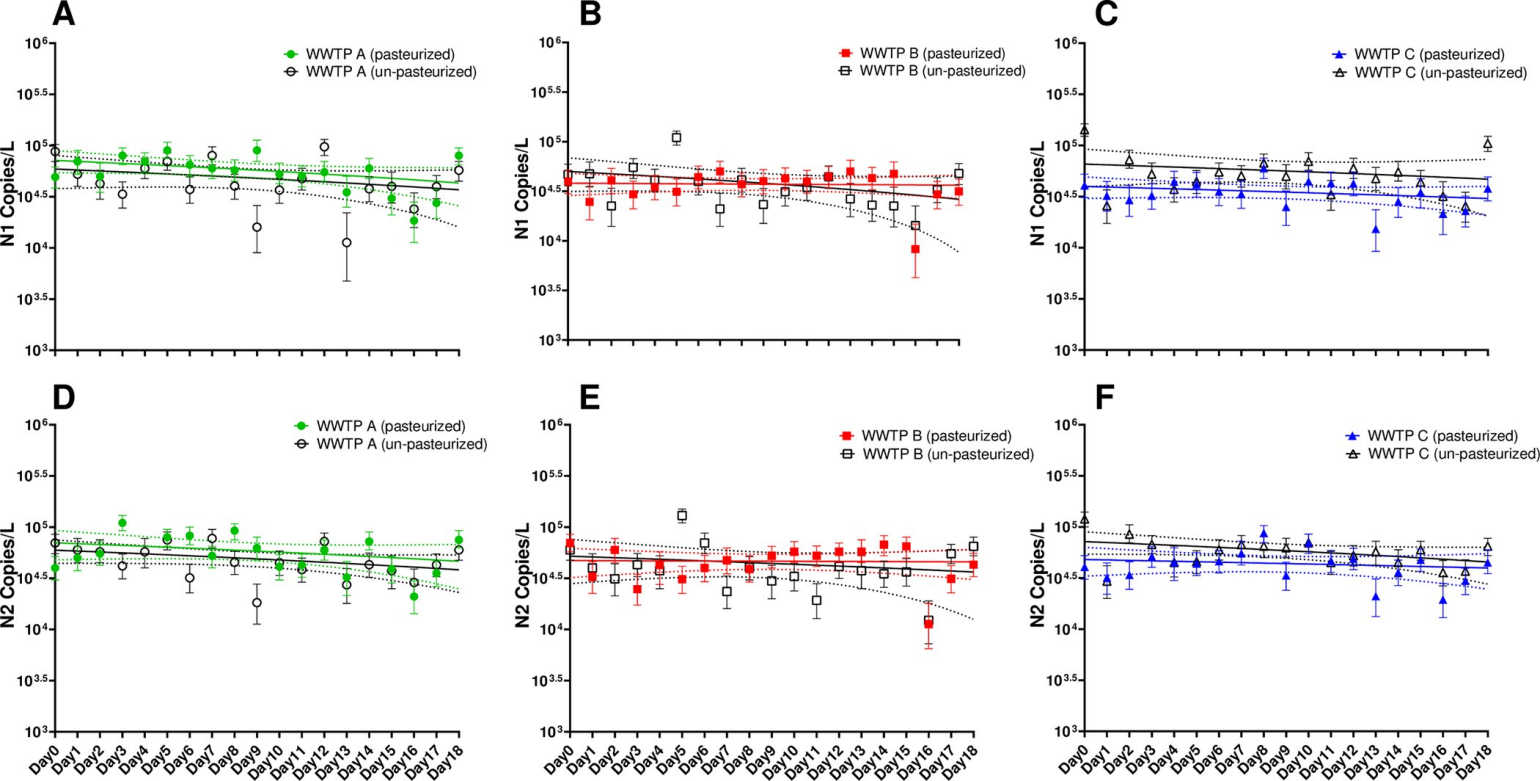
Concentration of SARS-CoV-2 N1 (A-C) and N2 (D-F) genes represented as copies/L in heat pasteurized and unpasteurized samples at three WWTP. Samples are represented by the mean concentration of a minimum of 20,000 droplets per sample +/- the upper and lower 95% confidence interval. Linear regressions of copies/L over the 19-day experiment in heat pasteurized and unpasteurized samples are represented by solid lines with the 95% confidence interval of the slope represented as dashed lines.

The concentration of SARS-CoV-2 N1 and N2 genes was measured in raw influent from three separate WWTP and field blank samples daily for 19 days (Fig 2). Heat pasteurized samples contained concentrations of N1 ranging from 8.25E + 03 to 9.00E + 04 copies/L and concentrations of N2 ranging from 1.13E + 04 to 1.10E + 05 copies/L while unpasteurized samples contained 1.43E + 04 to 1.43E + 05 and 1.23E + 04 to 1.30E + 05 copies/L of N1 and N2, respectively. The concentration of N1 and N2 in heat pasteurized and unpasteurized field blank samples was undetectable at all timepoints. At each of the three WWTP, the mean concentration of N1 and N2 in both heat pasteurized and unpasteurized samples did not vary significantly over time (*p*>0.05). This indicates that SARS-CoV-2 concentrations within wastewater collected for this study do not decay significantly when stored at 4°C for up to 19 days.

Heat pasteurization had no significant effect on the mean concentration of N1 and N2 copies/L in influent samples from Plant A and Plant B; however, heat pasteurized influent from Plant C contained significantly lower concentrations of both N1 and N2 compared to unpasteurized samples from the same location (p = 0.0039 and 0.0170, respectively). Although present, the difference in N1 and N2 concentrations in the heat pasteurized and unpasteurized samples from Plant C was less than half a log each. This result was supported by analyzing the effect of pasteurization on the total recovery control (Bovine Coronavirus, “BCoV”) across the three WWTP and field blank samples. Heat pasteurization did not result in significantly different mean concentrations of BCoV in the field blank, Plant A, or Plant B compared to their unpasteurized controls (p>0.05), but heat pasteurized samples from Plant C contained significantly lower mean concentrations of BCoV (p<0.0001) compared to the unpasteurized controls (0.58 log copies/L; S1 Fig in S1 File). This suggests that the heat pasteurization process impacts SARS-CoV-2 recovery based upon the influent sample matrix but does not diminish the ability to detect N1 and N2 within the sample

### Impact of sample source on viral decay and recovery

Individual WWTP receive variable matrix inputs due to a variety of factors including composition and size of the community served, sewage pipeline material, and local water hardness as well as any flushing procedures and industrial inputs. To assess the impact of matrix variability between distinct WWTP, the concentration of the total recovery control, BCoV, was compared with analysis of variance (ANOVA, S1 Fig in S1 File). As expected, total recovery in field blank samples was significantly higher (p<0.05) than any of the three WWTP in heat pasteurized and unpasteurized samples by an average of 0.19 log copies/L. No significant difference in BCoV concentrations was found within unpasteurized samples from the three WWTP; however, concentrations of BCoV in heat pasteurized samples from Plant C were significantly lower than concentrations at the other two WWTP (p<0.0001). Plant C is the smallest of the three WWTP in this study, serving a population size of approximately 67,000 and, on the day of sample collection, the wastewater collected was found to contain the lowest influent flow (8.65 MGD total, 129 G/person) and total suspended solids concentration compared to the other two WWTP we sampled (Table 1).

### Impact of freezing concentration filters on viral decay and recovery

**Fig 3:**
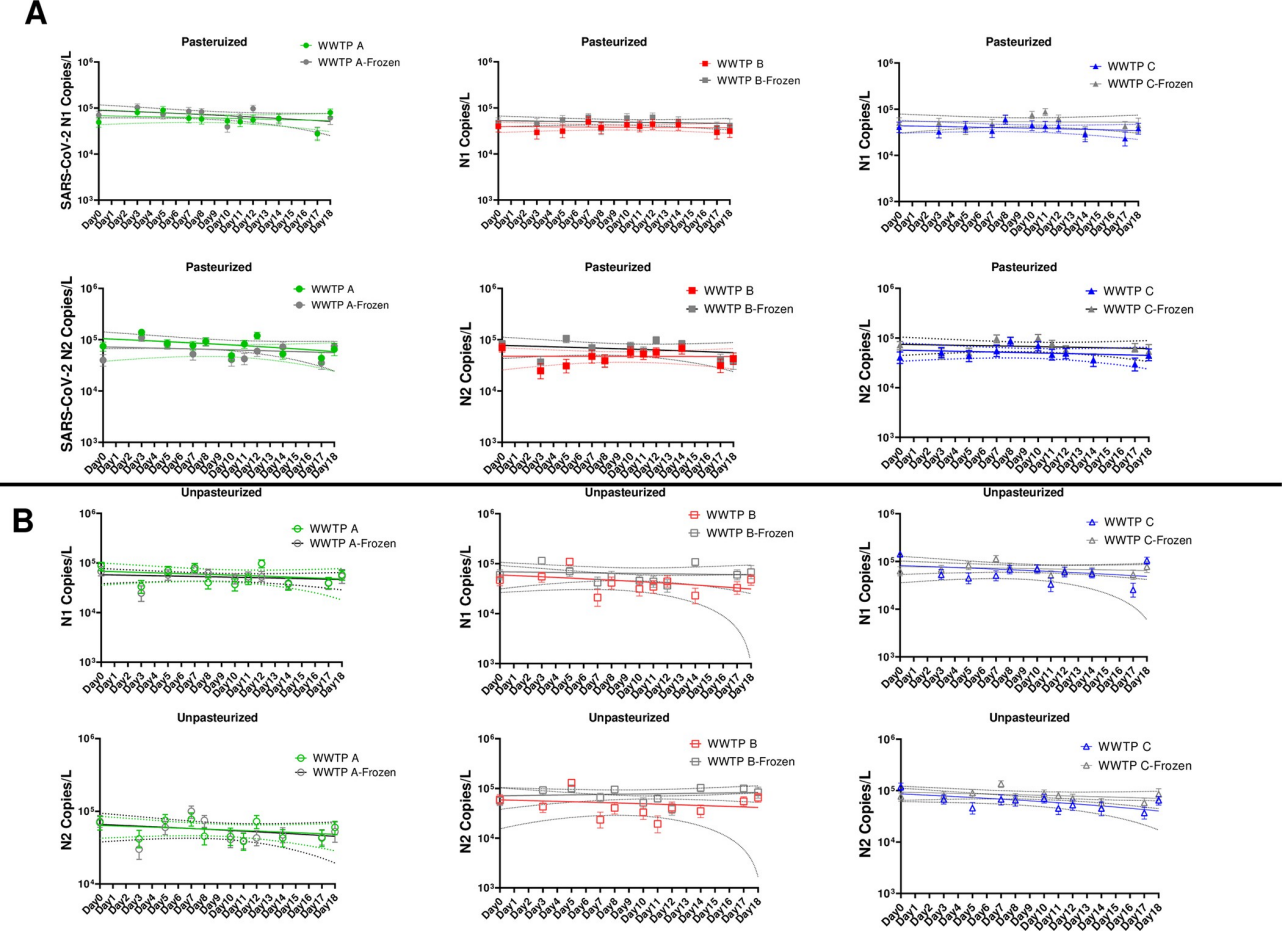
Concentration (copies/L) of SARS-CoV-2 N1 and N2 in heat pasteurized (A) and unpasteurized (B) samples at three WWTPs from filters frozen at -80°C compared to never frozen filters. Samples are represented by the mean concentration of a minimum of 20,000 droplets per sample +/- the upper and lower 95% confidence interval. Linear regressions of copies/L over the 18-day experiment are represented by solid lines with the 95% confidence interval of the slope represented as dashed lines. A subset of samples was used for this comparison to ensure the same analyst who performed the analysis on never frozen filters also performed the analysis on their frozen filter counterparts.

Due to time constraints or other laboratory-based limitations, processing influent samples from collection through quantification frequently requires more than one working day. Natural pause points are necessary to preserve sample integrity and viral signal, and one such pause point occurs immediately following sample concentration on filters. If necessary, filters containing the concentrated viral material can be frozen at -80°C immediately following filtration; however, the impact of this process on the viral signal is not well understood. Here, one replicate filter from each sample on each day was frozen at -80°C for processing at a later date (in this study, frozen filters were analyzed within one month). A subset of these samples was selected for analysis and comparison to the SARS-CoV-2 concentration data obtained from the filters processed day-of. Interestingly, the filters that were frozen at -80°C contained significantly higher concentrations of N1 and N2 at Plant B and Plant C in pasteurized samples and significantly higher concentrations of N2 at Plant B and Plant C in unpasteurized samples (paired *t*-test, Fig 3, S4 Table in S1 File) compared to the SARS-CoV-2 concentration obtained from filters that were immediately processed. However, the difference in concentrations between frozen and immediately processed filters was relatively small (less than 0.2 log copies/L). No significant differences in concentration were found between frozen and never frozen filters from Plant A.

### Sources of variability and noise in SARS-CoV-2 quantification

Molecular quantification of genetic targets requires multiple sample handling and processing steps which may introduce variability and noise. For complex procedures such as ddPCR, analyst training and experience may introduce significant amounts of noise within the data. To better understand if analyst experience level impacts concentration results, each step of the procedure from sample processing through SARS-CoV-2 quantification was assessed. In this study, an experienced analyst is defined as an individual who performs all steps of the molecular process (nucleic acid extraction through droplet generation) at least once a week for a minimum of 6 months with successful droplet generation at or above the threshold in at least 95% of samples analyzed. An inexperienced analyst is defined as an individual who has performed all steps of the molecular process as described above a minimum of three times but not more than 10 with variable successful droplet generation at or above the threshold.

**Fig 4:**
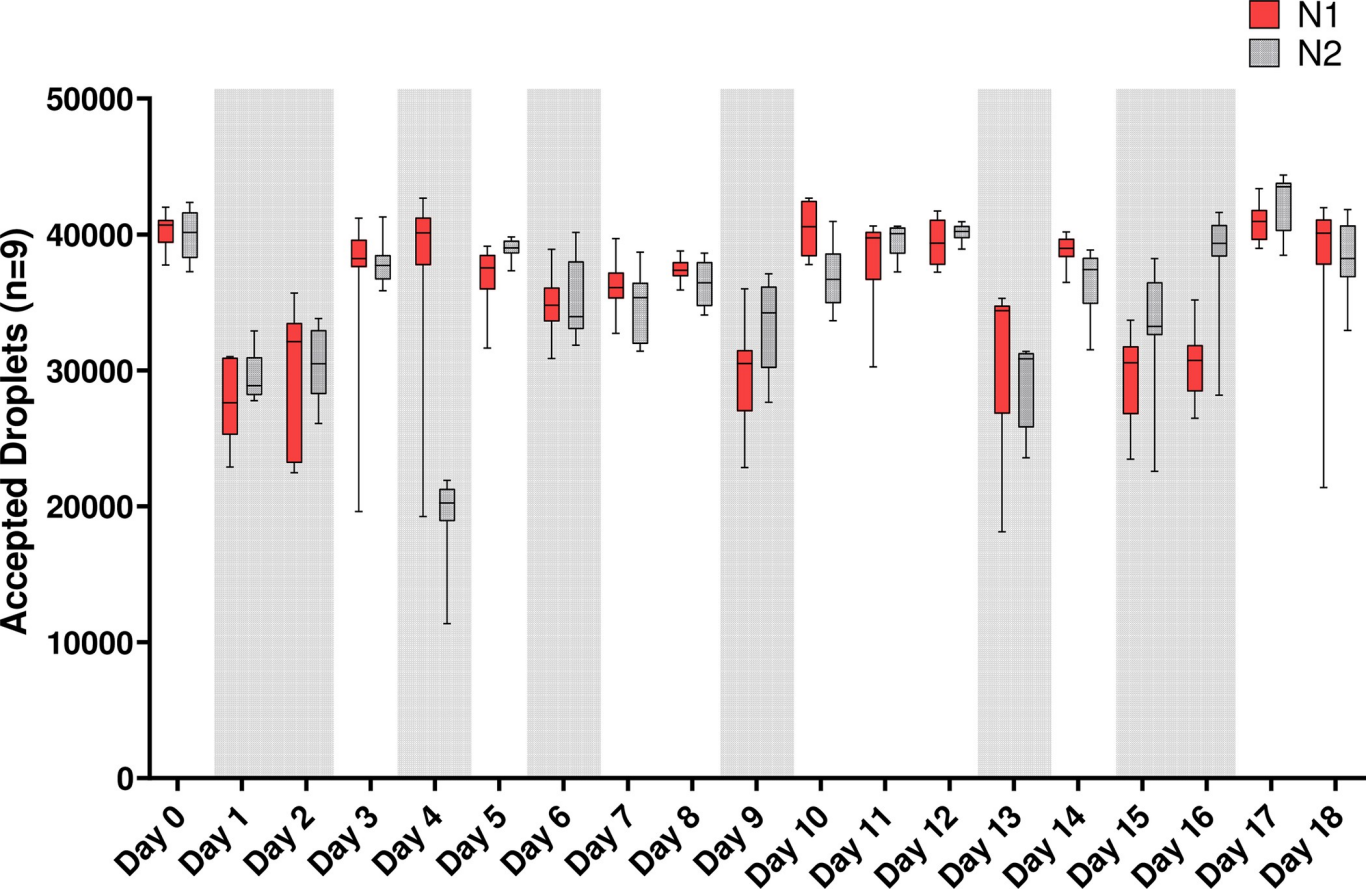
Box and whisker plots of the mean, minimum, and maximum of accepted droplets generated per genetic target (N1 or N2) per day across 9 samples (3 WWTP and 1 Field Blank heat pasteurized and unpasteurized for a total of 8 samples plus 1 no matrix control for a total of 9 samples analyzed). Days shaded in light gray represent data generated by a new analyst; unshaded days represent data generated by an experienced analyst.

Of the five parameters assessed (total viral recovery, extraction efficiency, RT efficiency, inhibition, and accepted droplet count), only accepted droplet count was significantly impacted by the analyst level of experience with more experienced analysts generating significantly more droplets (p<0.001, Fig 4). It should be noted that while analyst proficiency does impact the number of droplets produced, this does not affect the reported concentration of SARS-CoV-2 as the concentration will only be reported if the minimum droplet count threshold is met. Additionally, once the acceptable threshold for partitioning is met (in this study, 10,000 partitions per well), additional partitions (droplets) do not affect the final quantified concentration.

In addition to analyst experience level, other parameters measured also play a role in SARS-CoV-2 concentration variability. Distance-based redundancy analysis was used to identify if differences in RNA extraction efficiency, RT efficiency, and/or inhibition contributed to the variability observed in SARS-CoV-2 N1 and N2 concentration over the 19-day experiment. Extraction efficiency and RT efficiency both contributed to the variability in N1 concentration (p = 0.029 and 0.001, respectively) while RT efficiency contributed to the variability in N2 concentration (p = 0.001) of samples from the three WWTP. Although these two factors significantly impacted variability in SARS-CoV-2 concentration data, this effect did not diminish the ability to detect the genetic targets or the overriding trend indicating a lack of viral degradation over time. Interestingly, inhibition did not significantly impact concentration data variability. This is likely because the majority of the WWTP samples quantified were uninhibited during ddPCR (ranging from 68–100% inhibition control recovery).

## Discussion

Wastewater based epidemiology has been critical for the early detection and trend mapping throughout the COVID-19 pandemic. Monitoring the incidence and prevalence of disease at the population level using this approach has been critical for informing public health decisions. Despite the usefulness and predictive capabilities of WBE for viral disease outbreaks [2–4], inconsistency in surveillance methods and procedures has limited data comparisons between sewersheds and processing laboratories. Reducing sources of variability in quantification is crucial for effective WBE and allows for more accurate comparisons of disease dynamics in sewersheds worldwide during the current and future pandemics. Here, we assessed the concentration of SARS-CoV-2 in wastewater daily for 19 days to provide a high-resolution investigation of sources of variability in the WBE process.

Multiple factors impacting viral signal in wastewater influent can be controlled and optimized including sample storage (temperature, length of storage), handling (automated vs. hands-on time, analyst experience level), and processing (pasteurization, freeze-thaw cycles, quantification methods, etc.). In this study, we found that influent samples stored at 4°C maintained a detectable SARS-CoV-2 signal with no significant drop in concentration during the 19-day experiment regardless of whether or not the sample was heat pasteurized, indicating that the concentration of SARS-CoV-2 in wastewater is stable for more than two weeks if stored at 4°C. This result corroborates and expands upon data from other studies. Baldovin *et al*. [28] found that the SARS-CoV-2 concentration in influent samples from seven different WWTP did not degrade significantly when held at 4°C for 24 hours; however, their methodology differed from ours at the concentration step (ultrafiltration concentration vs. our electronegative membrane filtration), RT step (one step vs. our two step), and the platform used (qPCR vs. our ddPCR). Using a similar method to ours, Bivins *et al*. [29] also found that the SARS-CoV-2 signal is stable over 7 days when held at 4°C, regardless of whether the sample has been heat pasteurized. Together, these results suggest that sample storage at 4°C is a robust method allowing laboratories to store wastewater samples on site for up to two weeks without risk of SARS-CoV-2 signal degradation.

Heat pasteurization of wastewater influent samples is required at many universities and state laboratories to reduce any real or perceived risk of infectious SARS-CoV-2 transmission from the sample [30–32]. However, multiple studies report conflicting data regarding the impact of heat pasteurization on the SARS-CoV-2 signal in wastewater influent [29, 33]. Here, we found that heat pasteurization does not prevent the detection of SARS-CoV-2 N1 and N2 genes in samples collected from three distinct WWTP but does diminish the total recovery of the virus detected at a relatively small WWTP (Plant C). A thorough review by Ahmed *et al*. identified that the amount of total suspended solids and the wastewater matrix influence the occurrence of false negative errors that may result in non-detect of SARS-CoV-2 even when the virus is present [9]. Here, we found that heat pasteurization contributes to a significant decay of the virus in a WWTP with a small population size and low total suspended solids (Fig 2). Enveloped viruses, such as SARS-CoV-2, have been shown to have a high affinity for solids in wastewater 2 [34, 35], suggesting that the lower total suspended solids at Plant C may have impacted recovery after heat pasteurization even with minimal differences in concentration compared to the other two plants prior to pasteurization. The low concentration of total suspended solids measured at Plant C may also be related to the population size in the catchment which may impact sewer infrastructure. It is possible that the sewer system contributing to Plant C is compromised, allowing for increased inflow and infiltration thereby reducing the amount of total suspended solids and removing a preferential attachment site for SARS-CoV-2; however, this hypothesis would need to be tested in more detail. The influence of WWTP size and characteristics must be taken into consideration prior to processing. When wastewater samples are obtained from underserved regions with small population centers, the likelihood of false negative errors may increase, reducing the ability of public health officials to provide targeted intervention and mitigation measures to those regions. Additionally, the concentration method used prior to quantification may impact the viral concentration prior to or following pasteurization [36] and should be evaluated for its efficiency across WWTP.

Sample processing steps are time consuming, often requiring more than one full day from sample collection through quantification. Due to this, laboratories have considered multiple methods to store samples for varying durations of time to allow for batch processing; however, the impact of these methods on viral signal is not well understood. One such method involves freezing influent samples directly at -20°C or -80°C; however, multiple studies have shown that this type of freeze-thaw process significantly reduces the quantifiable viral signal [14, 29, 36]. Another method involves freezing the filters onto which the influent sample is concentrated. This method provides a natural pause point between two time-consuming steps (influent sample filtration/concentration and RNA extraction). Here, we assessed the impact of freezing on viral decay and recovery by comparing the SARS-CoV-2 concentration recovered from filters frozen at -80°C and filters that were processed immediately after concentration and never frozen. We found that freezing the filters does not reduce the viral signal compared to never frozen filters; in fact, for two of the three WWTP, the viral signal was slightly higher from frozen filters. One possible explanation for the result is the variability found in extraction and RT efficiency across samples in this study; however, further evaluation and comparison of these efficiencies in frozen and never frozen filters indicated no significant differences. The most likely explanation for this result is that freezing the filters at -80°C increases cell lysis in addition to the chaotropic lysis buffer added prior to nucleic acid extraction. This result indicates that freezing the filter concentrates at -80°C does not reduce the SARS-CoV-2 viral signal and may even increase the total recoverable signal due to additional cell lysis. This result requires further analysis but is promising for the recovery and quantification samples from WWTP with low concentration targets or highly variable matrices.

The influence of technician experience on highly technical steps was evident in the droplet generation step of ddPCR (Fig 4). Although less experienced technicians achieved the droplet counts required for target quantification, they exhibited higher within- and between-day variability, increasing the risk of unusable data. To reduce human error, automation of complex methods is frequently proposed and, where applicable, implemented [37]. However, automation for all procedures is not an option for many laboratories, and thus technician training is crucial to ensuring high quality quantification and recovery are achieved.

Interestingly, we found that the most significant sources of SARS-CoV-2 signal variability were factors that cannot be directly controlled including sample source matrix, extraction efficiency (if using an automated extraction method), and RT efficiency. The wastewater sample matrix is inherently complex and has frequently been cited as a potential limitation to WBE methods [1, 38]. While the concentration of the total processing control (BCoV) used in this study was not significantly different between sample sources over the entirety of the 19-day experiment, day-to-day variability was influenced by source. Additionally, sample source is the most likely contributor of differences in extraction and RT efficiencies which increased day to day variability. Other studies have found that sample matrix influences extraction and RT efficiencies in addition to the extraction kit components and RT enzyme used [39, 40]. During the current COVID-19 pandemic, most research efforts regarding RNA extraction efficiency and RT efficiency have focused on specific methods or kits rather than differences in efficiency introduced by the sample matrix itself [41, 42]. Additional research in this area is needed to better understand what components of the wastewater matrix influence these efficiencies to reduce day-to-day viral concentration variability.

This study has a few limitations. Influent samples were collected from three WWTP in the state of North Carolina over one 24-hour period. Although the WWTP are located in counties of differing population size and density these WWTP and associated sewer networks are relatively small and old, and influent samples from the sewersheds studied may not be globally generalizable. Extraction efficiency and RT efficiency were variable over the 19-day study which increases uncertainty in collected data. Regardless, the data presented in this study provide areas for consideration prior to implementing a WBE protocol to reduce variability and improve viral recovery allowing informed public health decisions during the current and future pandemics.

## Conclusions

By providing a high-resolution dataset assessing the impact of sample storage, handling, and technical ability on viral decay and recovery, results from this study can be used to guide best practices for reducing viral decay in wastewater samples used for WBE of pathogens. Our results indicate:

- SARS-CoV-2 does not degrade significantly in influent samples stored at 4°C for at least two weeks
- Heat pasteurization, a required step for many university and state laboratories, does not diminish the ability to detect viral signal in influent samples at WWTP serving large populations (>100,000 individuals), but alternative methods, such as filtration in a biological safety cabinet, should be considered to reduce false negative errors and increase viral recovery at small WWTP (<100,000 individuals) or WWTP with low concentrations of viral targets.
- If processing steps must be broken up over multiple days, filters used to concentrate wastewater samples may be stored at -80°C and processed within one month with no loss of viral signal.
- Analyst experience level may impact data generation during complex processing steps and should be closely monitored.

## Supporting information

S1 File Contains all supporting figures (S1 Fig), methods (S1 & S2 Methods), tables (S1-S4 Tables), and equations (S1 Equation). (DOCX)
