# Supplementary material for: Evaluating the impact of sample storage, handling, and technical ability on the decay and recovery of SARS-CoV-2 in wastewater
Source: PLoS One. 2022 Jun 24;17(6):e0270659. doi: 10.1371/journal.pone.0270659 (PMC9232146; doi:10.1371/journal.pone.0270659)
Supplement: S1 File — (DOCX) [file pone.0270659.s001.docx]

**Supplemental Information for manuscript titled:** Evaluating the impact of sample storage, handling, and technical ability on the decay and recovery of SARS-CoV-2 in wastewater

**Authors:** Rachelle E. Beattie, A. Denene Blackwood, Thomas Clerkin, Carly Dinga, Rachel T. Noble

**Number of Pages** (not including title page)**:** 10

**Number of Figures:** 1

**Number of Methods:** 2

**Number of Tables:** 4

**Number of Equations:** 1

**S1 Fig**. Concentration (copies/L) of BCoV (total processing control) in heat pasteurized (A) and unpasteurized (B) samples at three WWTPs and the field blank. Samples are represented by the mean concentration +/- the upper and lower 95% confidence interval. Linear regressions of copies/L over the 18-day experiment are represented by solid lines with the 95% confidence interval of the slope represented as dashed lines.


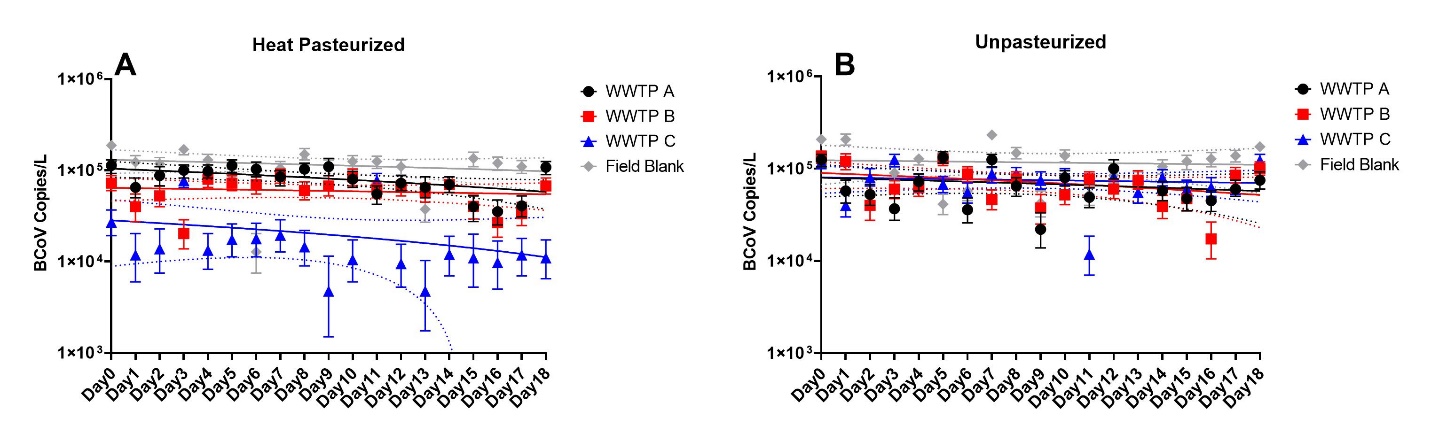


**S1 Method**. Total nucleic acid extraction from MCE filters including supplies, volumes, and procedure on the KingFisher™ Flex automated magnetic particle analyzer (Thermo Fisher Scientific, Waltham, MA) using the easyMag® NucliSENSE® reagents (bioMerieux, Durham, NC).

**Supplies and catalog number for total nucleic acid extraction**

| **Quantity** | **Description** | **Vendor** | **Catalog Number** |
| --- | --- | --- | --- |
| 1 | Tip comb | Fisher Sci. | 22-387-029 |
| 7 | 96 well, DWP | Fisher Sci. | 22-387-031 |
| 800µL | Wash Buffer 1 | BioMérieux | 280130 |
| 1000µL | Wash Buffer 2 | BioMérieux | 280131 |
| 500µL | Wash Buffer 3 | BioMérieux | 280132 |
| 50µL | Magnetic silica | BioMérieux | 280134 |
| 100µL | Buffer AE | Life Technologies | 19077 |

**Preparation of nucleic acid extraction plates**

- Lysate Plate
  - Dispense up to 950 µL of supernatant of lysed sample into a 96 Deep Well plate containing 50mL of magnetic beads.
    - Incubate 96 well Deep Well plate containing sample and Magnetic Beads for 10 minutes at room temperature.
- Wash 1 Plate
  - Dispense 400mL of Wash Buffer 1 into each sample well
- Wash 2 Plate
  - Dispense 400mL of Wash Buffer 1 into each sample well
- Wash 3 Plate
  - Dispense 500mL of Wash Buffer 2 into each sample well
- Wash 4 Plate
  - Dispense 500mL of Wash Buffer 2 into each sample well
- Wash 5 Plate
  - Dispense 500mL of Wash Buffer 3 into each sample well
- Eluate Plate
  - Dispense 100mL of AE Elution Buffer into each sample well
- Tip Comb Plate
  - Place 96DW tip comb into plate

Following 10 minute incubation of lysed sample + magnetic beads, insert plates into the Kingfisher™ Flex as directed by the program.

**Nucleic Acid Extraction Steps**

| 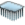 Tip1 | |  | | 96 DW tip comb | | |
| --- | --- | --- | --- | --- | --- | --- |
| 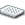 Pick-Up | | | Tip Comb |  | |  |
|  | | |  |  | |  |
| 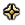 Binding 1 | | | Lysate 1 |  | |  |
| Beginning of step | | | Precollect | No | |  |
|  | | | Release time, speed | 00:00:20, Medium | |  |
| Mixing / heating: | | | Mixing time, speed | 00:03:20, Medium | |  |
|  | | | Heating during mixing | No | |  |
| End of step | | | Postmix | No | |  |
|  | | | Collect count | 5 | |  |
|  | | | Collect time [s] | 25 | |  |
| 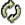 Collect Beads-1 | | | Lysate 1 |  | |  |
| Beginning of step | | | Precollect | No | |  |
|  | | | Release beads | No | |  |
| Mixing / heating: | | | Mixing time, speed | 00:02:30, Slow | |  |
|  | | | Heating during mixing | No | |  |
| End of step | | | Postmix | No | |  |
|  | | | Collect beads | No | |  |
| 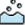 Wash 1 | | | Wash 1 |  | |  |
| Beginning of step | | | Precollect | No | |  |
|  | | | Release time, speed | 00:00:20, Medium | |  |
| Mixing / heating: | | | Mixing time, speed | 00:00:30, Medium | |  |
|  | | | Heating during mixing | No | |  |
| End of step | | | Postmix | No | |  |
|  | | | Collect count | 3 | |  |
|  | | | Collect time [s] | 2 | |  |
| 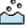 Wash 2 | | | Wash 2 |  | |  |
| Beginning of step | | | Precollect | No | |  |
|  | | | Release beads | Yes | |  |
| Mixing / heating: | | | Mixing time, speed | 00:00:30, Medium | |  |
|  | | | Heating during mixing | No | |  |
| End of step | | | Postmix | No | |  |
|  | | | Collect count | 3 | |  |
|  | | | Collect time [s] | 2 | |  |
| 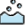 Wash 3 | | | Wash 3 |  | |  |
| Beginning of step | Precollect | No |  |  |  |  |
|  | Release beads | Yes |  |  |  |  |
| Mixing / heating: | Mixing time, speed | 00:00:30, Medium |  |  |  |  |
|  | Heating during mixing | No |  |  |  |  |
| End of step | Postmix | No |  |  |  |  |
|  | Collect count | 3 |  |  |  |  |
|  | Collect time [s] | 2 |  |  |  |  |

Protocol report

96DW_Flex_Water-Filtrate_Cefas_NucliSens_v3

10/12/2021 6:10:06 AM-07:00 2/2

| 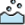 Wash 4 | Wash 4 |  |
| --- | --- | --- |
| Beginning of step | Precollect | No |
|  | Release beads | Yes |
| Mixing / heating: | Mixing time, speed | 00:00:15, Slow |
|  | Heating during mixing | No |
| End of step | Postmix | No |
|  | Collect count | 4 |
|  | Collect time [s] | 3 |
| 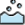 Wash 5 | Wash 5 |  |
| Beginning of step | Precollect | No |
|  | Release beads | Yes |
| Mixing / heating: | Mixing time, speed | 00:00:15, Slow |
|  | Heating during mixing | No |
| End of step | Postmix | No |
|  | Collect count | 4 |
|  | Collect time [s] | 3 |
| 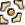 Elution | Elution |  |
| Beginning of step | Precollect | No |
|  | Release beads | Yes |
| Mixing / heating: | Mixing time, speed | 00:05:00, Medium |
|  | Heating temperature [°C] | 60 |
|  | Preheat | Yes |
| End of step | Postmix | No |
|  | Collect count | 4 |
|  | Collect time [s] | 3 |
| 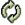 Collect Beads | Elution |  |
| Beginning of step | Precollect | No |
|  | Release beads | No |
| Mixing / heating: | Mixing time, speed | 00:01:30, Slow |
|  | Heating during mixing | No |
| End of step | Postmix | No |
|  | Collect beads | No |
| 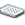 Leave | Tip Comb |  |

**S2 Method.** Optimization process for ddPCR assays.

For all ddPCR assays, optimization steps were as follows: each assay was initially analyzed in duplicate with gene standard (template as noted in the manuscript) concentrations between 5-200 copies/uL on a temperature gradient of 53-63℃. The optimal temperature was identified as the temperature with the best separation between positive and negative droplets with the least amount of “rain”. NTCs were included with each assay to evaluate the appropriate negative baseline for each gene. Next, assays were analyzed in duplex with their associated gene pair to evaluate performance, possible inhibition or cross reactivity, and specificity. All assays were found to have an optimal temperature of 55℃, thus this was used for the annealing temperature for assays in this study.

**S1.** Permitted flow, population size served, and 24-hour influent and effluent flow on the day of sample collection for the three WWTP in this study in metric units (cubic meters per day or CMD).

| **WWTP ID** | **Permitted Influent Flow (CMD)** | **Estimated Population Served**[16] | **24 Hour Influent Flow (CMD): August 9, 2021** | **24 Hour Effluent Flow (CMD): August 9, 2021** | **Total Suspended Solids (mg/L): August 9, 2021** |
| --- | --- | --- | --- | --- | --- |
| A | 7.95e4 | 151,589 | 5.98e4 | 5.67e4 | 253 |
| B | 2.80e5 | 550,000 | 1.78e5 | 1.73e5 | 288 |
| C | 3.97e4 | 67,743 | 3.27e4 | 5.51e4 | 156 |

**S2 Table.** Primer and probe sequences for SARS-CoV-2 and control assays with literature references to published sequences.

| **Target Gene** | ***Primers and Probes*** | ***Sequences 5'-3'*** | ***R*eference** |
| --- | --- | --- | --- |
| N1 Nucelopcapsid | nCoV N1 FWD | GACCCCAAAATCAGCGAAAT | 2019-Novel coronavirus (2019-nCoV) real-time rRT-PCR panel primers and probes. US Centers for Disease Control and Prevention. |
|  | nCoV N1 REV | TCTGGTTACTGCCAGTTGAATCTG |  |
|  | nCoV N1 FAM Probe | ACCCCGCATTACGTTTGGTGGACC |  |
| N2 Nucelocapsid | nCoV N2 FWD | TTACAAACATTGGCCGCAAA | 2019-Novel coronavirus (2019-nCoV) real-time rRT-PCR panel primers and probes. US Centers for Disease Control and Prevention. |
|  | nCoV N2 REV | GCGCGACATTCCGAAGAA |  |
|  | nCoV N2 FAMProbe | ACAATTTGCCCCCAGCGCTTCAG |  |
| Bovine Coronavirus | BCoV_FAM | CCTTCATATCTATACACATCAAGTTGTT | Decaro et al. Detection of bovine coronavirus using a TaqMan-based real-time RT-PCR assay. J Virological Methods 151 (2008) 167-171 doi:10.1016/j.jviromet.2008.05.016 |
|  | BCoV_F | CTGGAAGTTGGTGGAGTT |  |
|  | BCoV_R | ATTATCGGCCTAACATACATC |  |
| gyr A from haloalkaliphilic archaeon | Np_gyra_HEX | unpublished | Provided by Josh Steele, SCCWRP |
|  | Np_gyra_F | unpublished |  |
|  | Np_gyra_R | unpublished |  |
| Hepatitis G | HepF | CGGCCAAAAGGTGGTGGATG | Schlueter et al. 1996. Reverse transcription-PCR detection of hepatitis G virus. J. Clin. Microbiol. 34 (11):2660-2664. |
|  | HepR | CGACGAGCCTGACGTCGGG |  |
|  | Hep HEX Probe | AGGTCCCTCTGGCGCTTGTGGCGAG |  |
| Beta Actin | Mouse ACTB, 20X VIC | Proprietary, Life Technologies | |

**S3 Table.** Results of the Shaprio-Wilks tests for data normality.

|  | **N1** | | | | **N2** | | | |
| --- | --- | --- | --- | --- | --- | --- | --- | --- |
|  | Pasteurized | | Unpasteurized | | Pasteurized | | Unpasteurized | |
|  | Test statistic (W) | P value | Test statistic (W) | P value | Test statistic (W) | P value | Test statistic (W) | P value |
| WWTP A | 0.9674 | 0.7228 | 0.9412 | 0.3034 | 0.9662 | 0.7485 | 0.9575 | 0.555 |
| WWTP B | 0.9569 | 0.5423 | 0.9471 | 0.382 | 0.9664 | 0.7036 | 0.9523 | 0.4619 |
| WWTP C | 0.9723 | 0.8212 | 0.9235 | 0.149 | 0.9167 | 0.0983 | 0.9634 | 0.6961 |

**S4 Table.** Results of the unpaired t-test comparing N1 and N2 mean concentrations in WWTP sample processed immediately following filtration versus filters frozen at -80℃. Statistically significant values are bolded. The log difference between means is positive when the concentration of N1 or N2 is higher in samples that were processed immediately and negative if the concentration is higher from frozen filters.

| **Pasteurized** | | | | | | |
| --- | --- | --- | --- | --- | --- | --- |
|  | **N1** | | | **N2** | | |
| **Samples Tested** | ***t*** | **P value** | **Log Difference of Mean** | ***t*** | **P value** | **Log Difference of Mean** |
| WWTP A vs Frozen WWTP A | 1.023 | 0.3183 | -0.05953 | 1.437 | 0.1661 | -0.10008 |
| WWTP B vs Frozen WWTP B | 3.061 | **0.0062** | **-0.11058** | 2.138 | **0.0450** | **-0.14455** |
| WWTP C vs Frozen WWTP B | 2.478 | **0.0223** | **-0.13646** | 2.145 | **0.0445** | **-0.12717** |
| **Unpasteurized** | | | | | | |
|  | **N1** | | | **N2** | | |
| **Samples Tested** | ***t*** | **P value** | **Log Difference of Mean** | ***t*** | **P value** | **Log Difference of Mean** |
| WWTP A vs Frozen WWTP A | 0.6506 | 0.5227 | 0.041839 | 0.0778 | 0.9338 | 0.004714 |
| WWTP B vs Frozen WWTP B | 1.832 | 0.0819 | -0.15878 | 2.411 | **0.0257** | **-0.19183** |
| WWTP C vs Frozen WWTP B | 0.5605 | 0.5814 | -0.04103 | 2.117 | **0.0470** | **-0.11927** |

**S1 Equation.** Equation to calculate gene copies/L.

To calculate X gene copies/L, the machine generated copies/µL (A) is multiplied by the total volume of the duplicated PCR reaction (here, 25 µL/ reaction * 2 = 50 µL) divided by the volume of cDNA in the PCR reaction (here, 10 µL), multiplied by the RNA to cDNA dilution factor (here, 2), multiplied by the total nucleic extraction elution volume (here, 100 µL), divided by the volume filtered (here, 40 mL), multiplied by the mL to L conversion factor.

$$\text{X }\frac{copies}{L} = \text{A}\frac{copies}{\mu L} \times\frac{50 \mu L}{10 \mu L} \times2 \times100 \mu L \div40 mL \times\frac{1000 mL}{1 L}$$
